# Supplementary material for: A Diluted Electrolyte for Long-Life Sulfurized Polyacrylonitrile-Based Anode-Free Li-S Batteries
Source: Polymers (Basel). 2022 Aug 15;14(16):3312. doi: 10.3390/polym14163312 (PMC9413679; doi:10.3390/polym14163312)
Supplement: Supplementary file 1 [file polymers-14-03312-s001.zip › polymers-1872345-supplementary.pdf]

Supporting Information

Ting Ma[1,2], Xiuyun Ren[3], Liang Hu[4], Wanming Teng[1,2], Xiaohu Wang[2,5], Guanglei Wu[3], Jun Liu[2], Ding Nan[1,2]*, Baohua Li[6], Xiaoliang Yu[4]*

[1] College of Chemistry and Chemical Engineering, Inner Mongolia University, Hohhot, 010021, China
[2] Inner Mongolia Key Laboratory of Graphite and Graphene for Energy Storage and Coating, School of Materials Science and Engineering, Inner Mongolia University of Technology, Hohhot, 010051, China
[3] College of Materials Science and Engineering, Qingdao University, Qingdao
266071, China
[4] Department of Mechanical Engineering, Research Institute for Smart Energy, The Hong Kong Polytechnic University, Hong Kong, China
[5] Rising Graphite Applied Technology Research Institute, Chinese Graphite Industrial Park-Xinghe, Ulanqab, Inner Mongolia, 013650, China
[6] Shenzhen Key Laboratory on Power Battery Safety and Shenzhen Geim Graphene Center, Tsinghua Shenzhen International Graduate School (SIGS), Shenzhen, 518071 China

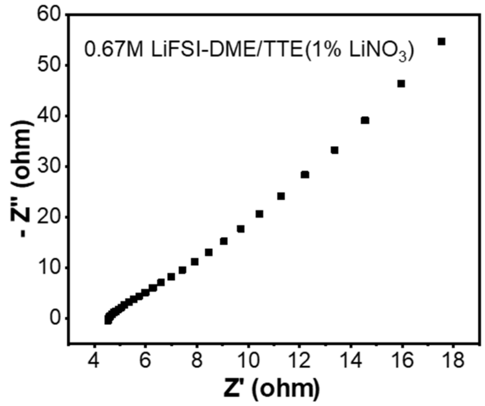

Figure S1 EIS spectra of symmetrical cells (stainless steel/separator/stainless steel) using 0.67 M LiFSI–DME/TTE ($LiNO_3$).

The ionic conductivity of electrolyte corresponds to the migration ability of lithium ions in the electrolyte and plays a key role in the battery performance, especially the multiplicity performance. We assembled stainless steel symmetric cells to test the electrochemical impedance spectra of both electrolytes. The ionic conductivity σ can be calculated by the equation $\sigma = d/RS$, where d is the diaphragm thickness of 25 μm, S is the contact area of 2 cm^2 and R is the impedance. The impedance of the electrolyte was 4.5 Ω, and the conductivity was calculated to be 2.78×10^{-4} S cm^{-1}.

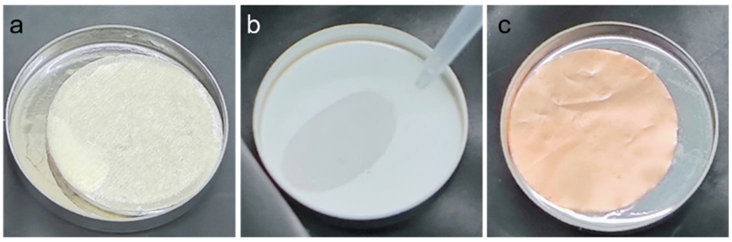

Figure S2 Optical images showing wettability of 0.67 M LiFSI-DME/TTE ($LiNO_3$) on lithium foil (a), separator (b), and copper foil (c).

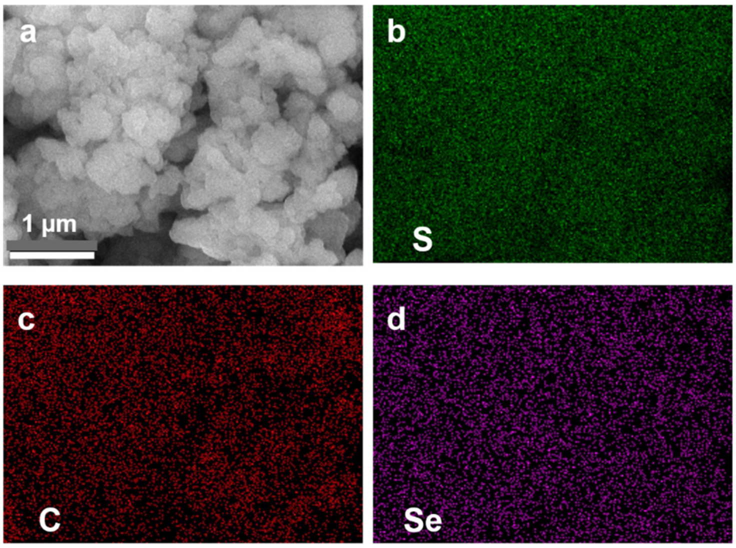

Figure S3 EDS elemental mapping images of the pPAN/SeS_2 composite for (b) sulfur, (c) carbon and (d) selenium.

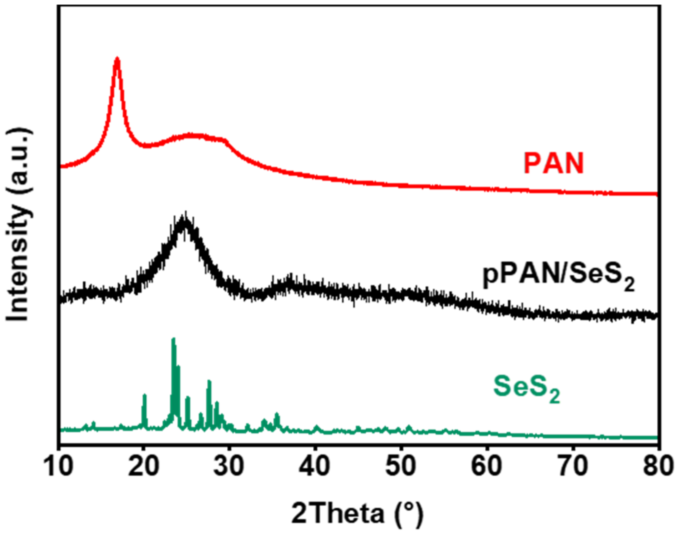

Figure S4 XRD of pPAN/SeS_2 (black), PAN (red), SeS_2 (green).
